# Diagnostic value of basic and extended procedures in pediatric fever of unknown origin – results of a nation-wide surveillance study

**DOI:** 10.1186/s40348-026-00249-w

**Published:** 2026-06-30

**Authors:** Mira Katarina Bienioschek, Alice Lejeune, Gonza Ngoumou, Michael S. Urschitz, Stefanie Dollinger, Martina Hagenberg, Kai Lehmberg, Simone Schrauth, Johannes Wolf, Horst von Bernuth, Dirk Foell, Tilmann Kallinich

**Affiliations:** 1 Department of Pediatric Respiratory Medicine, Immunology and Critical Care Medicine, Charité Universitätsmedizin Berlin, Augustenburger Platz, Berlin, 13353 Germany; 2 Charité Universitätsmedizin Berlin, Charité Competence Center for Traditional and Integrative Medicine (CCCTIM), Berlin, Germany; 3 German Paediatric Surveillance Unit (GPSU), Division of Paediatric Epidemiology, Institute of Medical Biostatistics, Epidemiology and Informatics, University Medical Centre of the Johannes Gutenberg- University, Mainz, Germany; 4 German Centre for Paediatric and Adolescent Rheumatology, Garmisch-Partenkirchen, Germany; 5 Children’s Hospital St. Elisabeth and St. Barbara, Halle (Saale), Germany; 6 Department of Pediatric Hematology and Oncology, University Medical Center Eppendorf, Hamburg, Germany; 7 Department of Paediatrics, Division of Paediatric Infectious Diseases, University Hospital of Würzburg, Würzburg, Germany; 8 St. Vincenz-Krankenhaus Paderborn, Klinik für Kinder- und Jugendmedizin, Paderborn, Germany; 9 Department of Pediatric Rheumatology and Immunology, University Hospital Münster, Münster, Germany; 10 German Center for Child and Adolescent Health (DZKJ), partner site Berlin, Berlin, Germany; 11 Deutsches Rheuma-Forschungszentrum (DRFZ), an Institute of the Leibniz Association, Berlin, Germany

**Keywords:** Fever of unknown origin, Children, Diagnostic, Infectious, Inflammatory, Neoplastic

## Abstract

**Background:**

Pediatric fever of unknown origin (FUO) remains a major diagnostic challenge encompassing infectious, autoimmune and autoinflammatory as well as malignant diseases. Despite improved diagnostical procedures, no underlying disease can be identified in approximately one third of patients. Current guidelines recommend stepwise diagnostical approaches based on potential diagnostic clues (PDC+). This prospective nation-wide surveillance study aims to evaluate basic and advanced diagnostic procedures in the diagnostic of FUO.

**Methods and results:**

Via the German Pediatric Surveillance Unit, children were pseudonymized enrolled based on the following inclusion criteria: fever ≥ 38.5 °C for at least five out of ten consecutive days and no identifiable cause after standard diagnostic workup. The questionnaire collected patient data, symptoms, performed diagnostic workup, and the final diagnosis, if applicable.

Among 113 included children, an underlying disease was identified in 72 cases (63.7%). 26 patients (23.1%) had infections, 31 children (27.4%) had systemic onset juvenile idiopathic arthritis/Still’s disease (sJIA/SD), and 15 children (13.3%) were classified as “other diagnoses” including autoinflammatory diseases, vasculitides, and malignancies. Children with sJIA/SD presented with more clinical symptoms (mean = 4.6, SD = 2.17) compared to those with infections (mean = 2.7, SD = 1.40, *p*=.002). Basic diagnostic workup revealed at least one PDC + in every case (mean = 6.6). Most PDC+ (mean = 3.1, SD = 1.82) were identified by history taking and physical examination as well as by laboratory testing and blood count (mean = 2.5 PDC+, SD = 1.32). A total of 370 basic imaging investigations, e.g. abdominal ultrasound and echocardiography, provided in total 50 PDCs+ demonstrating their high diagnostical yield. Primarily in children with unclear clinical presentation advanced imaging revealed PDC+. Overall, the number of misleading PDCs (PDC-) was low (mean = 1.0, SD = 1.14) with autoantibody testing being the basic diagnostic procedure accounting for the majority of PDC-.

**Conclusion:**

The results of this nation-wide surveillance study highlight the value of basic and advanced diagnostical approaches in the management of pediatric FUO. Careful history taking, physical examination, and targeted basic diagnostic testing can identify multiple PDC+ with only few misleading PDC-, which may contribute to diagnostic accuracy and avoid unnecessary testing. The findings can be introduced into guidelines in order to standardize and improve the diagnostic approaches in children with FUO.

**Supplementary Information:**

The online version contains supplementary material available at 10.1186/s40348-026-00249-w.

## Background

Fever of unknown origin (FUO) is a challenging clinical syndrome typically caused by rare or oligosymptomatic underlying diseases [1]. FUO was originally defined as recurrent fever exceeding 38.3 °C lasting for at least three weeks with no identified cause after one week of hospital-based investigations [2]. More recent pediatric studies defined FUO by a duration varying between seven and 21 days without the mandatory need of inpatient investigations [1, 3].

Identifying the cause of FUO is crucial for appropriate therapy and to avoid long-term complications from the underlying diseases. A rational diagnostic work-up is essential to minimize misleading, false-negative test results, to avoid unnecessary and invasive procedures and to preserve resources. In adults with FUO, several studies analysed structured diagnostic protocols [4–7]. Previously abnormalities in clinical signs, laboratory results, or imaging findings were defined as “potential diagnostic clues” (PDCs) leading to the diagnosis or the performance of further targeted follow-up tests [4]. Based on these observations, the authors developed diagnostic approaches emphasizing the step-by-step implementation of basic and extended diagnostic measures [5].

The structured approach was also incorporated into a national guideline extrapolating evidence from adult to pediatric populations [8]. In this framework basic diagnostic measures comprise detailed history, physical examination, standard laboratory and basic imaging studies. Depending on the presence or absence of PDCs, further diagnostic tests are recommended (see Supplementary Table 1) [8].

Using data from a nation-wide prospective surveillance study this work analyses the spectrum of underlying diseases causing pediatric FUO in Germany. We aim on evaluating the diagnostical value of a defined set of basic and extended diagnostical measures in order to support the development of structured evidence-based diagnostical algorithms for pediatric FUO.

## Methods

### Aim, design and setting of the study

Incident cases fulfilling the following criteria were collected by the German Paediatric Surveillance Unit (GPSU) between May 2016 and December 2018: (i) rectally measured temperature of ≥ 38.5 °C on at least 5 out of 10 consecutive days, and (ii) no identifiable cause of fever despite the consideration of medical history, clinical examination, general laboratory testing, infectiological and imaging examinations. Children and adolescents with primary or secondary immunodeficiency or immunosuppression were excluded.

### Procedures and data collection

The GPSU surveys approximately 90% of all pediatric hospitals in Germany (*n* = 264) on a monthly basis. In cases of reported FUO, a questionnaire was sent to the treating physicians. To ensure anonymity of the reporting institutions and cases, the returned questionnaires were pseudonymized by the GPSU.

The questionnaire collected information on demographics, general condition, prior medical history, travel history, previous fever episodes and fever characteristics. Fever patterns were classified as continuous, intermittent (1–2 fever spikes per day), or episodic (repeated fever-free intervals of at least one day).

Clinical symptoms, physical findings, laboratory parameters and imaging results were documented (Supplementary Table 1). Physicians had the option to send a sera sample to the University Hospital Münster for analysis of S100 A8/A9 protein levels.

Physicians were asked whether a diagnosis was established after day 10 of fever and which findings contributed to the diagnosis. The questionnaire also contained details on response of empirical treatment.

Symptoms and findings were reviewed by the four authors MB, TK, AL and GN and rated as positive (PDC+) or negative PDC (PDC-) (see Supplementary Table 2). A PDC + was classified as a clinical sign or test result from the basic diagnostic workup that is typically associated with the final diagnosis based on guidelines and textbooks. In contrast, PDC- were findings that did not correspond with the final diagnosis. Abnormalities revealed through extended diagnostics were classified as ePDC. Patients were categorized into four groups: infections, sJIA/SD, other diagnoses (OD), and no diagnosis.

### Statistical analysis

Data from the returned questionnaires were entered into a Microsoft Excel spreadsheet and analyzed using IBM SPSS Statistics 31. Nominal variables were evaluated via cross-tabulations and χ² tests, including pairwise comparisons with Bonferroni correction for multiple comparisons. Continuous variables were analyzed descriptively and compared within the diagnostic groups using the nonparametric Kruskal-Wallis test.

Three binary logistic regression analyses were performed to evaluate associations between clinical variables and diagnostic outcomes. Receiver Operating Characteristics (ROC) curves were used to visualize diagnostic test accuracy using area under the curve (AUC) as a measure of classification performance.

Statistical significance was defined as *p* ≤ .05, with correction for multiple testing using the Bonferroni method (α = 0.0083). Due to the exploratory design, p-values are to be interpreted as hypothesis-generating.

Visualizations were created using Microsoft Excel, SPSS Statistics 31, diagrams.net, Python and SankeyMATIC for flow diagrams.

## Results

### Patient characteristics

Out of 179 returned questionnaires, 113 cases fulfilled the inclusion criteria for children presenting with FUO and had sufficient data sets. 66 questionnaires were excluded due to insufficient or incomplete data and missed inclusion criteria. In 18 cases (15.9%) anonymized medical reports were additionally available.

The average age of the patients was 6.6 years (SD = 5.28), with no significant differences in age and sex distribution between the groups (*p*=.065 and 0.184, respectively).

Logistic regression revealed an association between being male and a lower probability of being diagnosed with sJIA/SD (OR = 0.23; 95% CI: 0.07–0.72; *p*=.012).

### Underlying identified diseases causing FUO

**Fig. 1:**
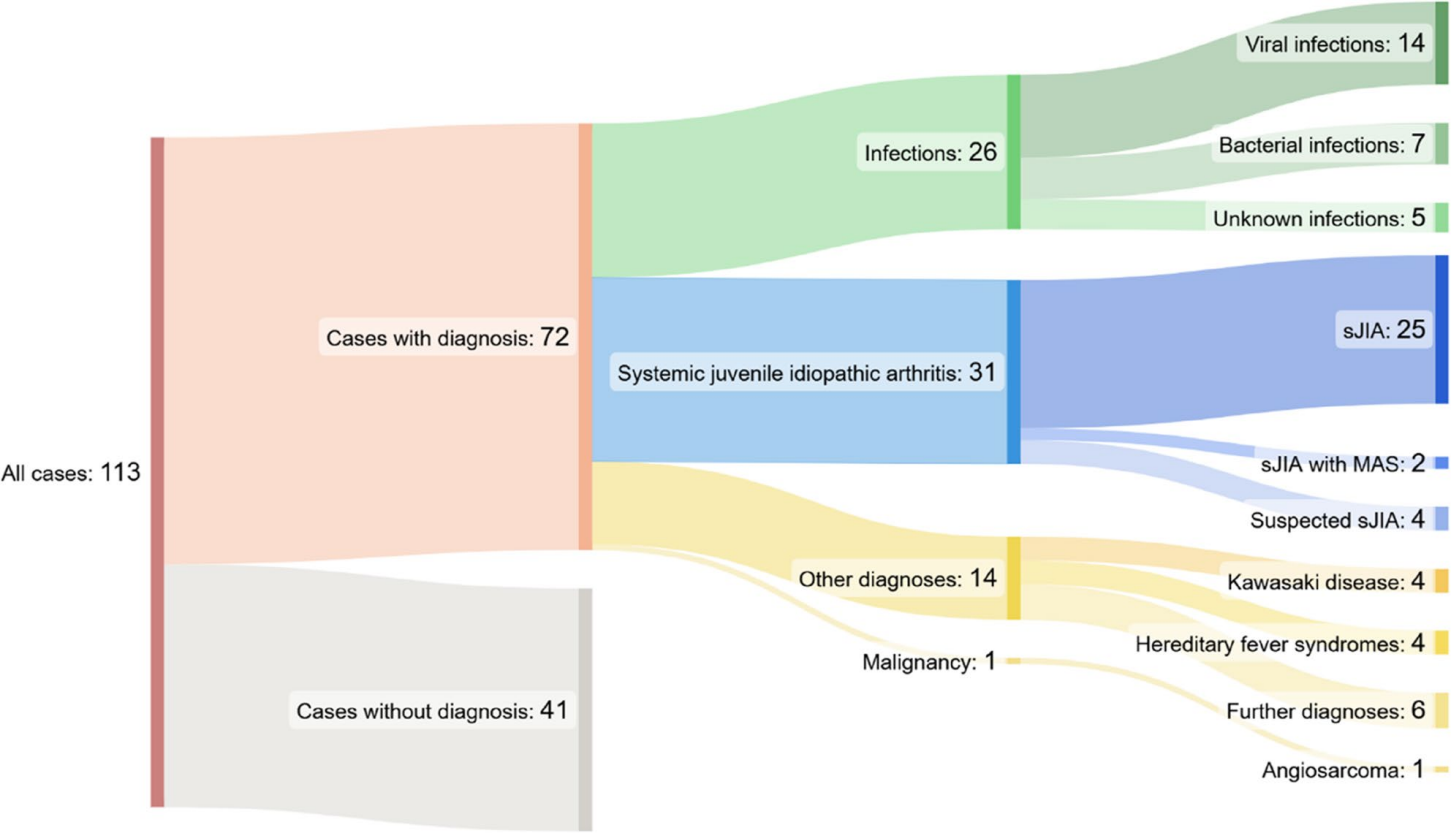
Number of diagnosed and undiagnosed patients and the distribution of underlying causes (Based on [9])

In 72 cases (63.7%) a diagnosis was confirmed: 26 children (23.0%) had an infection, 31 (27.4%) had systemic juvenile idiopathic arthritis/Stills disease (sJIA/SD) and 14 (12.4%) were grouped under OD together with one malignancy case (0.9%) (Fig. 1). The OD category includes various autoinflammatory diseases and hereditary fever syndromes (Table 1). 41 children (36.3%) remained undiagnosed.

**Table 1:** Distribution of Diagnoses in the Clustered Study Population and Pre-existing Conditions

Cluster	Diagnosis	Preexisting Conditions
Infections	meningitis	prematurity
	Cytomegalovirus (CMV) meningoencephalitis	
	2x CMV infection	1x trisomy 21 1x SGA neonate with recurrent bronchitis and swallowing disorder
	2x viral infection, unspecified	
	2x pyelonephritis	1x Hirschsprung’s disease
	2x adenovirus infection	1x prematurity
	2x Epstein-Barr virus infection	
	2x Coxsackie virus infection	
	urinary tract infection	
	endocarditis	aortic valve defect
	renal abscess	
	typhoid fever	
	yersiniosis	
	influenza	
	pneumonia	
	mastoiditis	
	parvovirus B19 infection	
	pleuropneumonia	prematurity
	visceral leishmaniosis	
	herpes simplex virus encephalitis	
sJIA/SD	25x systemic juvenile idiopathic arthritis/Still’s disease (sJIA/SD)	1x streptococcal-associated arthritis, 1x VSD, Hashimoto’s thyroiditis, chronic urticaria, 1x epilepsy, developmental delay, pulmonary valve stenosis
	2x sJIA/SD with macrophage activation syndrome	1x atopic eczema
	4x suspected sJIA/SD	
Other Diagnoses	2x suspected Kawasaki disease (KD), incomplete KD 1x KD	
	1x Incomplete KD	febrile seizure
	1x familial mediterranean fever (FMF)	
	1x TRAPS 1x heterozygous TRAPS and CAPS	1x mitral insufficiency, partial anomalous pulmonary venous return
	1x febrile infection-related epilepsy syndrome (FIRES)	
	1x Blau syndrome	
	1x sarcoidosis	
	1x systemic Lupus erythematosus (SLE)/mixed connective tissue disease	thalassemia minor
	1x seronegative polyarthritis	
	1x secondary hemophagocytic lymphohistiocytosis	trigeminal neuralgia
	1x Crohn’s disease	
	1x hepatic angiosarcoma	

*CAPS *Cryopyrin-associated periodic syndrome, *CMV *Cytomegalovirus, *FIRES* febrile infection-related epilepsy syndrome, *KD *Kawasaki disease, *TRAPS* TNF receptor-associated periodic syndrome, *SGA* small for gestational age, *sJIA/SD* systemic juvenile idiopathic arthritis/Still’s disease, *VSD* ventricular septal defect

Pre-existing conditions were reported in 25 patients (22.1%) (Table 1). History taking revealed travel to Spain and Portugal in a child diagnosed with visceral leishmaniasis.

### Clinical features and tests discriminating disease groups

Intermittent fever was the most common fever pattern observed in 72 children (63.7%), particularly in sJIA/SD (*n* = 25/31; 80.6%) and OD (*n* = 11/15; 73.3%). Continuous fever occurred in 24 children (21.2%), episodic fever in 12 (10.6%) and recurrent fever in one child (0,9%) without apparent intergroup differences (*p*=.145).

The mean peak body temperature was 40.0 °C, temperatures did not differ between the groups (*p*=.814).

Patients with sJIA/SD presented with more clinical symptoms (mean = 4.61,SD = 2.17) than those with infections (mean = 2.73, SD = 1.40, *p*=.002) or without a diagnosis (mean = 2.46, SD = 1.83, *p*<.001). Children with OD had a mean of 3.67 symptoms (SD = 1.59).

**Fig. 2:**
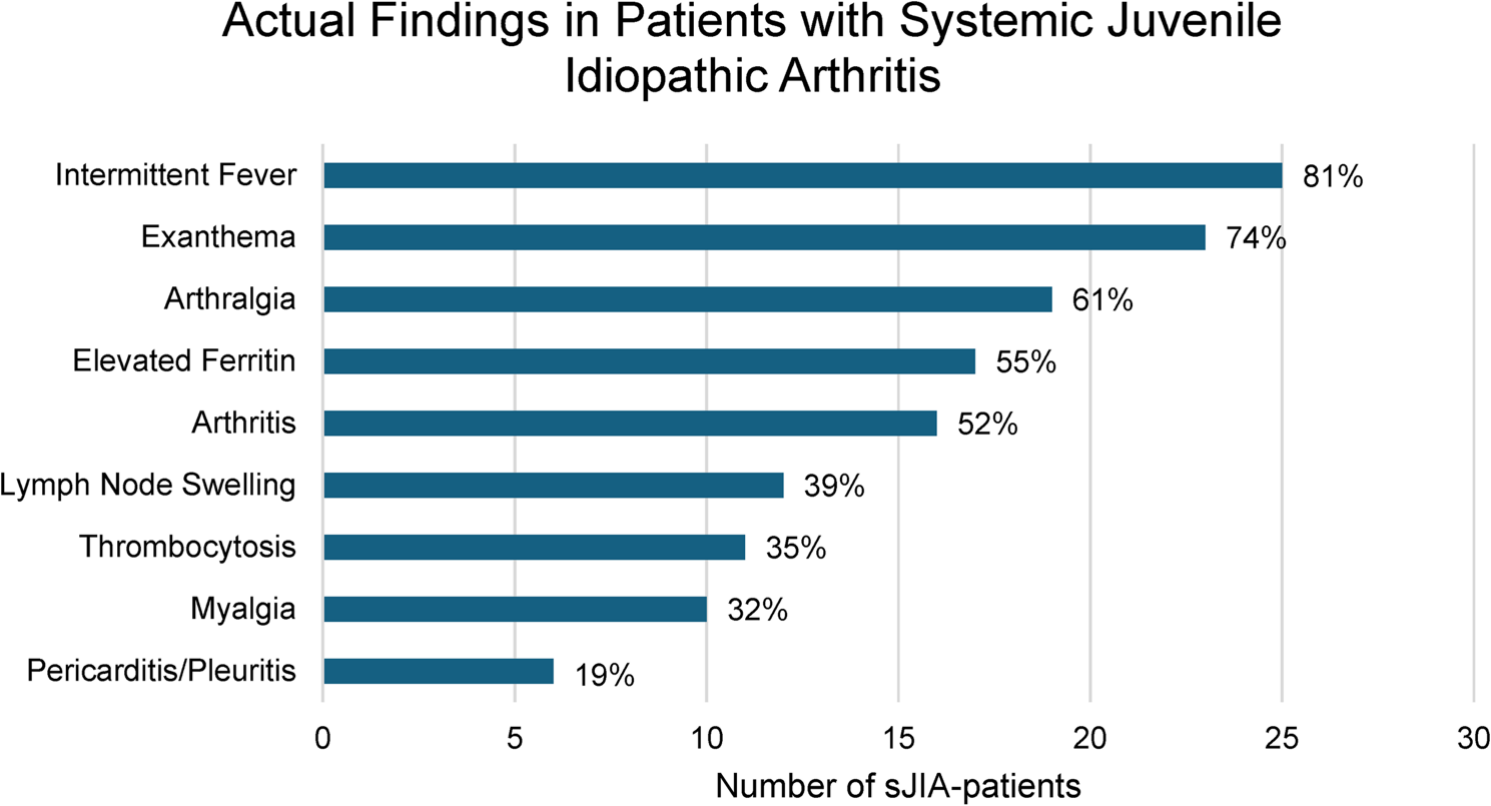
Actual findings in patients with sJIA/SD (*n* = 31)

Symptoms characteristic for sJIA/SD (Fig. 2), such as exanthema, arthritis, arthralgia, and myalgia, were less common in other disease groups. A binary logistic regression revealed arthralgia being strongly associated with a sJIA/SD diagnosis (OR = 12.4; 95%-CI:3.60-43.04; *p*<.001). In contrast, the prevalence of lymphadenopathy, a symptom linked to sJIA/SD, was not different when compared to other groups (*p*=.465).

Conjunctivitis was observed in four patients with OD (*n* = 4/15; 26.7%), mainly in cases of Kawasaki disease (KD), and more frequently observed than in infections (3.8%; *p*=.031) and undiagnosed cases (2.4%; *p*=.005). Logistic regression suggested that conjunctivitis was associated to higher odds of OD (OR = 5.15; 95% CI: 1.19–22.19; *p*=.028). Age and sex showed no association, and the model was not statistically significant overall (*p*=.136), with low discriminatory power in the ROC analysis for conjunctivitis (AUC = 0.603; 95%-CI: 0.43–0.77; *p*=.201).

In contrast, children with sJIA/SD showed elevated ferritin concentrations (mean = 3905.9 µg/l, SD = 8338.68) compared to those with infections (mean = 357.9 µg/l; SD = 605.80, *p*=.011) or without a diagnosis (430.5 µg/l; SD = 633.39 *p*=.011).

Additionally, levels of lactate dehydrogenase (LDH) were higher in patients with infections (mean = 444.4 U/l, SD = 180.52) than in undiagnosed patients (mean = 216.8 U/I, SD = 1187.68, *p*=.028). Mean LDH levels were 370.2 U/l (SD = 145.35) in sJIA/SD and 322.1 U/l (SD = 141.98) in OD.

S100A8/A9 sera protein was highest in sJIA/SD patients (mean = 99,206.5 ng/ml, SD = 85646.55), followed by infections (mean = 9730.0 ng/ml, SD = 7339.77), OD (mean = 21,105.0 ng/ml), and undiagnosed cases (mean = 3741.0 ng/ml, SD = 7481.97, *p*=.337).

The overall C-reactive protein (CRP) level was 94.2 mg/l (SD = 82.21), with no significant differences across groups (*p* = .123), similar to other laboratory parameters including blood count parameters.

### Use of diagnostic procedures

A total of 2,017 basic and 200 extended diagnostic procedures were registered (mean = 19.6, SD = 5.82, Table 2). Children with infections underwent fewer diagnostic procedures (mean = 16.4, SD = 5.78) compared to those with sJIA/SD (mean = 20.6; SD = 4.94, *p*=.004) and OD (mean = 21.3; SD = 6.11, *p*=.009). Undiagnosed children received an average of 20.3 (SD = 5.77) procedures.

**Table 2:** Overview of the Quantity of Diagnostic Procedures in the study cohort

	Infections	sJIA/SD	Other diagnoses	No diagnosis	Total	*P* Value (KW)
Patients (No.)	26	31	15	41	113	
Total diagnostic procedures (No.)	425	639	320	833	2217	0.007
Total diagnostic procedures, mean (SD)	16,4 (5.78)	20,6 (4.94)	21,3 (6.11)	20,3 (5.77)	19,6 (5.82)	
Laboratory, microbiological and immunological investigations						*P* (χ²)
Blood count, No. (%)	25 (96.2)	30 (96.8)	15 (100.0)	39 (95.1)	109 (96.5)	0.075
Laboratory panel, No. (%)	25 (96.2)	31 (100.0)	15 (100.0)	39 (95.1)	110 (97.3)	0.128
Urine test, No. (%)	23 (88.5)	26 (83.9)	14 (93.3)	39 (95.1)	102 (90.3)	0.423
Urine culture, No. (%)	13 (50.0)	15 (48.4)	10 (66.7)	18 (43.9)	56 (49.6)	0.513
Blood culture, No. (%)	24 (92.3)	26 (83.9)	12 (80.0)	38 (92.7)	100 (88.5)	0.425
Stool culture, No. (%)	12 (46.2)	19 (61.3)	12 (80.0)	29 (70.7)	72 (63.7)	0.105
Sputum culture, No. (%)	3 (11.5)	3 (9.7)	3 (20.0)	9 (22.0)	18 (15.9)	0.462
Skin swab, No. (%)	5 (19.2)	2 (6.5)	4 (26.7)	2 (4.9)	13 (11.5)	0.059
Mucosal swab, No. (%)	12 (46.2)	15 (48.4)	5 (33.3)	14 (34.1)	46 (40.7)	0.543
Lumbar puncture, No. (%)	6 (23.1)	4 (12.9)	5 (33.3)	6 (14.6)	21 (18.6)	0.310
RT23 test, No. (%)	3 (11.5)	7 (22.6)	3 (20.0)	7 (17.1)	20 (17.7)	0.741
Interferon-release assay, No. (%)	3 (11.5)	12 (38.7)	4 (26.7)	9 (22.0)	28 (24.8)	0.118
Occult blood, No. (%)	0 (0)	6 (19.4)	1 (6.7)	9 (22.0)	16 (14.2)	0.052
Fecal calprotectin, No. (%)	5 (19.2)	12 (38.7)	5 (33.3)	20 (48.8)	42 (37.2)	0.108
Autoantibodies, No. (%)	12 (46.2)	25 (80.6)	11 (73.3)	35 (85.4)	83 (73.5)	0.003**
Imaging						*P* (χ²)
Chest X-ray, No. (%)	16 (61.5)	27 (87.1)	13 (86.7)	37 (90.2)	93 (82.3)	0.017
Abdominal ultrasound,, No. (%)	22 (84.6)	31 (100.0)	14 (93.3)	38 (92.7)	105 (92.9)	0.165
Joint ultrasound, No. (%)	0 (0)	13 (41.9)	3 (20.0)	2 (4.9)	18 (15.9)	< 0.001***
Echocardiography, No. (%)	17 (65.4)	30 (96.8)	14 (93.3)	35 (85.4)	96 (85.0)	0.007**
Electrocardiogram, No. (%)	7 (26.9)	19 (61.3)	10 (66.7)	22 (53.7)	58 (51.3)	0.030
Advanced diagnostics						*p* (χ²)
MRI, No. (%)	7 (26.9)	6 (19.4)	5 (33.3)	11 (26.8)	29 (25.7)	0.763
CT/PET-CT, No. (%)	2 (7.7)	1 (3.2)	1 (6.7)	1 (2.4)	5 (4.4)	0.724
Additional ultrasounds, No. (%)	2 (7.7)	0 (0)	0 (0)	0 (0)	2 (1.8)	0.078
TEE, No. (%)	1 (3.8)	0 (0)	0 (0)	0 (0)	1 (0.9)	0.337
Electroencephalogram, No. (%)	1 (3.8)	0 (0)	0 (0)	0 (0)	1 (0.9)	0.337
Genetics, No. (%)	0 (0)	1 (3.2)	3 (20.0)	0 (0)	4 (3.5)	0.002**
Histology, No. (%)	1 (3.8)	0 (0)	3 (20.0)	1 (2.4)	5 (4.4)	0.015*
Protein S100 A8/A9, No. (%)	3 (11.5)	20 (64.5)	3 (20.0)	5 (12.2)	31 (27.4)	< 0.001***
Bone marrow puncture, No. (%)	4 (15.4)	11 (35.5)	4 (26.7)	8 (19.5)	27 (23.9)	0.281
Serology, No. (%)	23 (88.5)	22 (71.0)	13 (86.7)	37 (90.2)	95 (84.1)	0.133

*CT* computer tomography, *KW * Kruskal-Wallis test, *MRI * magnet resonance imaging, *PET-CT * positron emission tomography-computed tomography, *RT23 * tuberculin skin test, *SD * standard deviation, *sJIA/SD * systemic juvenile idiopathic arthritis/Still’s disease, *TEE * transoesophageal echocardiography

In more than 85% of the children standard blood and laboratory tests, urine dipstick analysis, blood cultures, abdominal ultrasound, and echocardiography were performed. Autoantibody and echocardiography testing were less frequently performed in infections (*p*=.003, *p*=.007) and joint ultrasound was more commonly performed in sJIA/SD (*p*<.001).

In 67 diagnosed children (93.1%) and 38 undiagnosed children (92.7%) advanced diagnostic procedures (Supplementary Table 1) were used (*p*=.080).

Serological testing was the most common advanced procedure performed in 84.1% of patients. MRI was the most frequently used advanced imaging technique, used in 29.9% patients with infections, 19.4% with sJIA/SD, 33.3% with OD and 26.8% without a diagnosis (*p*=.763). In total, 29 patients received 14 whole body and 20 organ-specific MRI examinations.

### Potential diagnostic clues

**Fig. 3:**
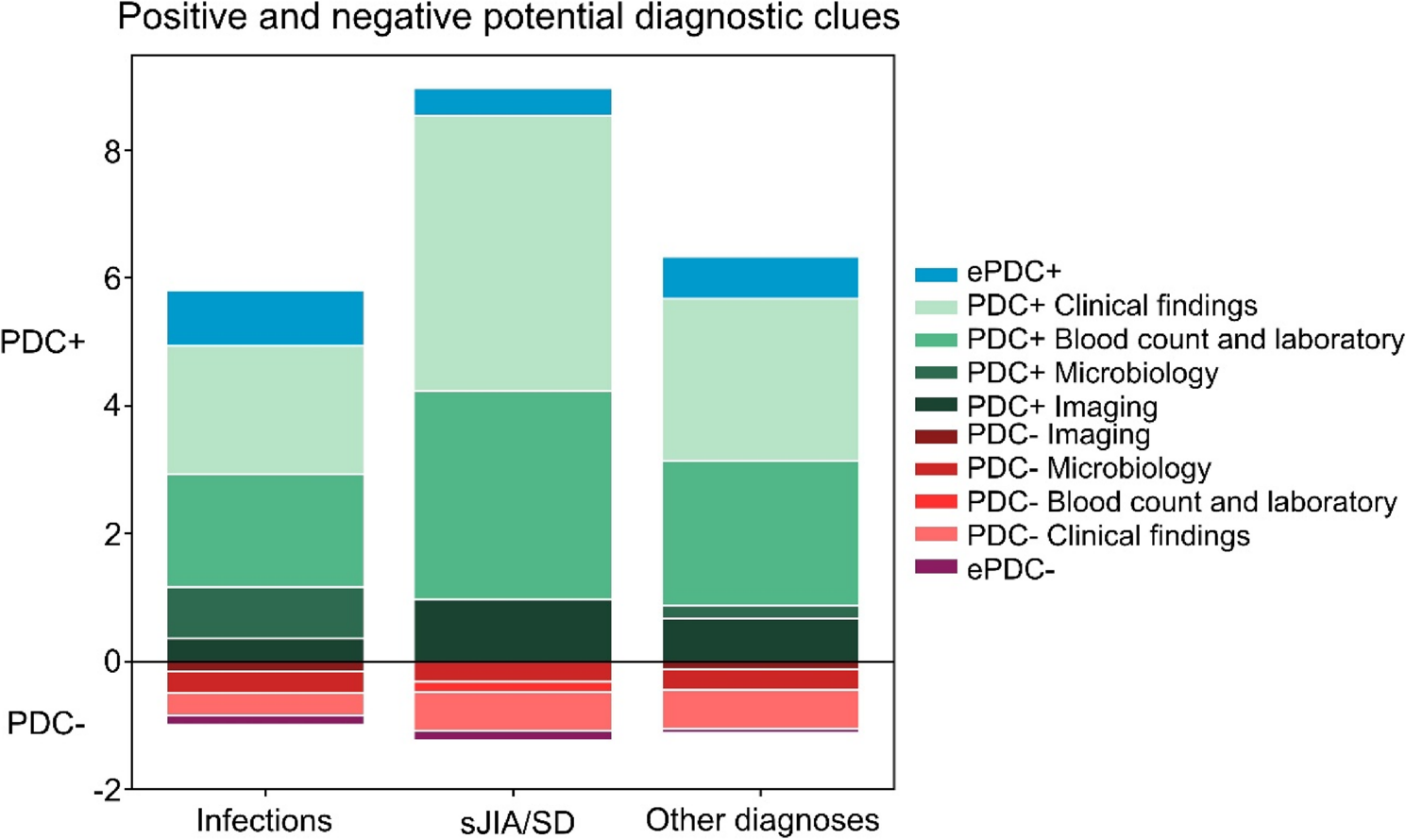
Mean values of positive and negative PDC (PDC+/-) through basic and extended diagnostics (ePDC)

Basic diagnostic procedures generated 478 PDC+, with every diagnosed patient showing at least one, while number of PDC- was low (*n* = 33). Among the average of 6.64 PDC+ identified per patient, most derived from clinical examination and history-taking (mean = 3.11 PDC+). Laboratory investigations generated 2.51 PDC + per patient (Fig. 3; Table 3).

**Table 3:** Positive (PDC+) and Negative (PDC-) Potentially Diagnostic Clues in the total population and subgroups of the clustered study population

	Infections (*n* = 26)	sJIA/SD (*n* = 31)	Other Diagnoses (*n* = 15)	Total (*n* = 72)	*P* Value
PDC+ from all basic diagnostic measures, mean (IQR)	4.92 (2)	8.55 (3)	5.67 (3)	6.64 (4)	< 0.001***
PDC- from all basic diagnostic measures, mean (IQR)	0.85 (1)	1.10 (2)	1.07 (2)	1.00 (2)	0.622
Clinical Findings					
PDC+, mean (IQR)	2.00 (2)	4.32 (3)	2.53 (1)	3.11 (2)	< 0.001***
PDC-,, mean (IQR)	0.35 (0)	0.61 (1)	0.60 (1)	0.50 (1)	0.298
Blood Count and Laboratory					
PDC+, mean (IQR)	1.77 (1)	3.26 (2)	2.27 (2)	2.51 (1)	< 0.001***
PDC-, mean (IQR)	0.00 (0)	0.16 (0)	0.00 (0)	0.07 (1)	0.062
Microbiology					
PDC+, mean (IQR)	0.81 (1)	0 (0)	0.20 (0)	0.33 (1)	< 0.001***
PDC-, mean (IQR)	0.35 (1)	0.32 (1)	0.33 (0)	0.33 (1)	0.806
Imaging					
PDC+, mean (IQR)	0.35 (1)	0.97 (2)	0.67 (1)	0.68 (1)	0.007*
PDC-, mean (IQR)	0.15 (0)	0.00 (0)	0.13 (0)	0.08 (0)	0.035*
Findings from Further diagnostics					
ePDC+, mean (IQR)	0.85 (0)	0.42 (1)	0.67 (1)	0.63 (1)	0.028*
ePDC-, mean (IQR)	0,15 (0)	0.16 (0)	0.07 (0)	0.14 (0)	0.431

*IQR*   interquartile range, *PDC * positive potentially diagnostic clue

Subgroups: Infections, sJIA/SD and other diagnoses

**Fig. 4:**
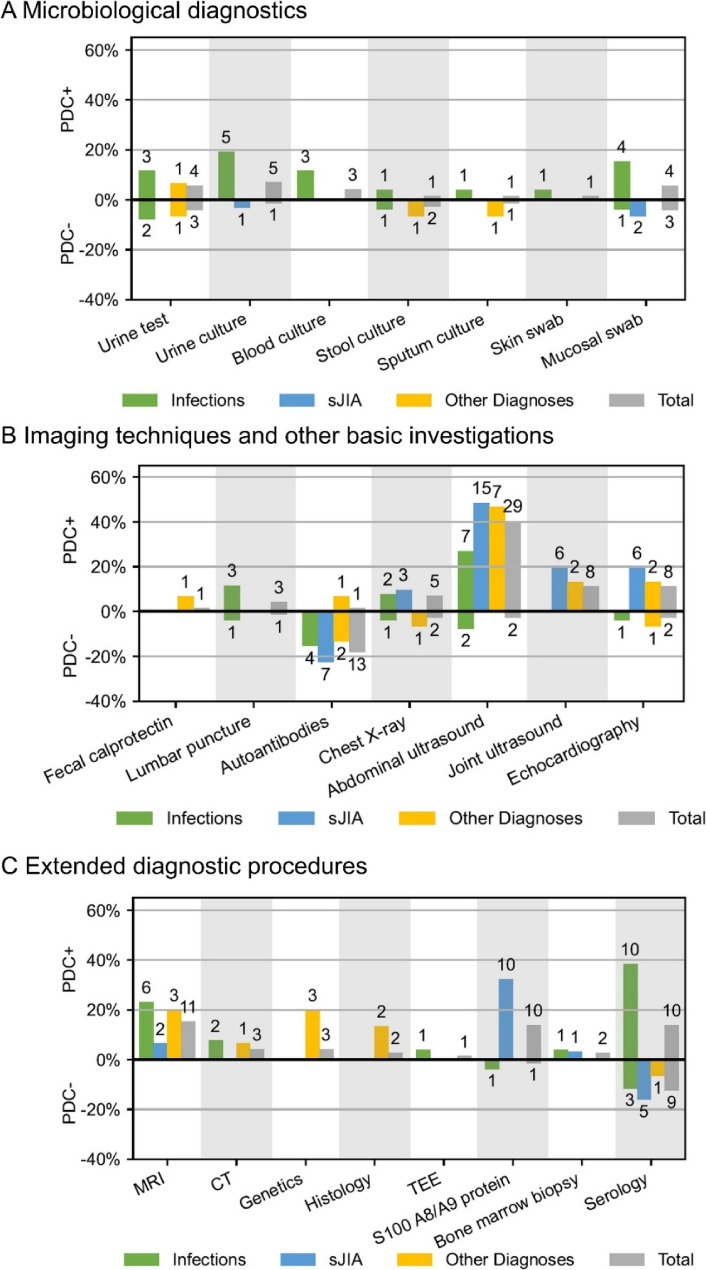
Positive and negative PDC in basic diagnostic procedures: **A** Microbiological testing, **B** Microbiological and imaging procedures, **C** Extended diagnostics.CT – computed tomography, MRI – magnet resonance imaging

Imaging studies contributed multiple PDC+, with abdominal ultrasound producing the most PDC+ (29 PDC + vs. 2 PDC-) (Fig. 4b; Table 3, supplementary Table 2). Three chest X-rays provided PDC + in two children with infections, while two were misleading (Supplementary Table 2). Echocardiography was informative in eight children. Joint ultrasound was mainly useful in sJIA/SD patients. (Fig. 4b).

Urine cultures yielded PDC + in five cases but were inconsistent in one sJIA/SD patient. Abnormal dipstick urine test identified three infections and one case of leukocyturia in KD, but were misleading in three cases (Fig. 4a, supplementary Table 2).

Mucosal swabs detected adenovirus-, Epstein-Barr virus- (EBV)- and influenza-nucleic acid in four children and bacterial growth in two, while three results were misleading (Fig. 4a, supplementary Table 2).

Blood cultures were informative in three patients (endocarditis, typhoid infection, candidemia). Salmonella typhi was also detected in a stool culture. Stool cultures were misleading in two patients (Fig. 4a). Sputum samples identified CMV-DNA, but were misleading in a TRAPS patient. One wound swab was PDC+, identifying Streptococcus pyogenes in a child with mastoiditis with empyema. Fecal calprotectin yielded one PDC+, indicating Crohn’s disease.

Lumbar punctures were informative in three children with infection (two meningitis, one Herpes simplex virus (HSV) encephalitis). Positive detection of glucose in CSF yielded one PDC- in a child with a renal abscess.

Autoantibody testing contributed to the diagnosis in one child with systemic lupus erythematosus, but was misleading in 13 children including seven sJIA/SD patients. ANA antibodies were the most frequently detected misleading PDC in 10 patients, whereas c-ANCA, ENA, and ACPA were each present in one patient (see Table 4, Supplementary Table 2).

**Table 4:** Overview of number of helpful (PDC+) and misleading (PDC-) potential diagnostic clues in our cohort

Pat.	Diagnosis	(e)PDC+/(e)PDC-					
		Clinical findings	Blood count & Laboratory	Micro-biology	Imaging	Auto-antibodies	Extended diagnostics
Inf1	Meningitis	1+/1-	0/0	1/0	0/-1	0/0	2/-1
Inf2	CMV meningoencephalitis	3+/0	0/0	1/0	1/0	0/0	1/0
Inf3	CMV infection	0/0	0/0	1/0	0/0	0/0	1/0
Inf4	Viral infection	5+/0	2/0	1/0	1/0	0/0	1/0
Inf5	CMV infection	2+/-1	2/0	2/0	0/0	0/0	0/-1
Inf6	Acute pyelonephritis	1+/0	1/0	2/0	1/0	0/0	1/0
Inf7	Pyelonephritis	1+/-2	2/0	2/0	1/0	0/0	0/-1
Inf8	Adenovirus infection	3+/0	2/0	1/0	0/0	0/0	0/0
Inf9	Adenovirus infection	4+/0	2/0	1/0	0/0	0/0	0/0
Inf10	Urinary tract infection	0/0	1/0	1/0	0/0	0/0	0/0
Inf11	EBV tonsillitis	3/0	2/0	1/0	0/0	0/-1	0/0
Inf12	EBV infection	2/0	2/0	0/0	0/0	0/0	1/0
Inf13	Coxsackie virus infection	2/0	2/0	0/0	0/0	0/-1	1/0
Inf14	Coxsackie virus	2/0	1/0	0/-1	0/0	0/-1	1/0
Inf15	Endocarditis	2/0	1/0	1/0	0/0	0/0	1/0
Inf16	Renal abscess	1/0	3/0	1/-1	1/0	0/0	1/0
Inf17	Typhoid fever	4/0	2/0	2/0	1/0	0/0	0/0
Inf18	Yersiniosis	0/0	2/0	0/0	0/0	0/-1	1/0
Inf19	Influenza, candidemia	2/0	3/0	2/-1	0/0	0/0	1/0
Inf20	Pneumonia	2/-2	3/0	0/0	1/-2	0/0	0/0
Inf21	Mastoiditis, empyema, SVT	1/0	1/0	1/0	0/0	0/0	3/0
Inf22	Respiratory viral infection	3/0	2/0	0/0	0/0	0/0	1/-1
Inf23	Parvovirus B19	3/0	2/0	0/-1	0/-1	0/0	1/0
Inf24	Pleuropneumonia	1/-3	2/0	0/0	1/0	0/0	1/-1
Inf25	Visceral leishmaniosis	2/0	4/0	0/0	1/0	0/0	1/-1
Inf26	HSV encephalitis	2/0	2/0	1/0	0/0	0/0	1/0
S1	sJIA/SD	4/0	4/0	0/0	1/0	0/0	1/0
S2	sJIA/SD	2/-2	4/0	0/0	1/0	0/0	0/0
S3	sJIA/SD	6/0	3/0	0/0	0/0	0/0	1/0
S4	sJIA/SD	6/0	2/0	0/0	1/0	0/0	0/-1
S5	sJIA/SD	4/0	3/0	0/0	0/0	0/0	0/0
S6	sJIA/SD	6/0	3/0	0/0	1/0	0/-1	0/0
S7	sJIA/SD	6/0	5/-1	0/0	2/0	0/0	0/0
S8	sJIA/SD	7/-2	2/0	0/0	1/0	0/0	0/0
S9	sJIA/SD	5/0	4/0	0/0	1/0	0/0	0/0
S10	sJIA/SD	4/-1	3/0	0/0	1/0	0/0	0/0
S11	sJIA/SD	5/0	5/0	0/-1	1/0	0/0	0/0
S12	sJIA/SD	1/-1	4/0	0/0	0/0	0/0	0/0
S13	sJIA/SD	6/0	2/-1	0/-1	1/0	0/-1	1/-1
S14	sJIA/SD	4/-1	3/0	0/0	0/0	0/0	1/0
S15	sJIA/SD	5/0	2/0	0/0	0/0	0/0	1/0
S16	sJIA/SD	4/-1	3/-1	0/0	2/0	0/0	0/0
S17	sJIA/SD	1/0	4/0	0/0	0/0	0/0	1/0
S18	sJIA/SD	7/0	3/0	0/0	1/0	0/0	1/0
S19	sJIA/SD	6/-1	4/0	0/0	1/0	0/0	1/0
S20	sJIA/SD	1/-1	5/0	0/0	0/0	0/0	0/0
S21	sJIA/SD	3/0	2/0	0/0	2/0	0/-1	0/0
S22	sJIA/SD	4/0	1/0	0/-1	2/0	0/-1	1/0
S23	sJIA/SD	3/-1	4/0	0/0	2/0	0/0	1/-1
S24	sJIA/SD with MAS	6/0	3/-1	0/0	1/0	0/-1	1/-1
S25	sJIA/SD	7/-2	3/0	0/0	1/0	0/0	1/-1
S26	sJIA/SD with MAS	3/0	4/-1	0/0	2/0	0/0	1/0
S27	sJIA/SD	6/-3	2/0	0/0	3/0	0/0	0/0
S28	Suspected sJIA	3/0	2/-1	0/0	0/0	0/-1	0/0
S29	Suspected sJIA	3/-1	3/0	0/0	1/0	0/0	0/0
S30	Suspected sJIA	3/-1	5/0	0/0	0/0	0/-1	0/0
S31	Suspected sJIA	3/0	4/0	0/0	0/0	0/0	0/0
AD1	Kawasaki disease	3/-1	1/0	0/0	1/0	0/0	0/0
AD2	Kawasaki disease	4/0	4/0	1/0	1/0	0/0	0/0
AD3	Incomplete Kawasaki disease	1/-1	3/0	0/0	0/0	0/0	0/0
AD4	Kawasaki disease	2/0	2/0	0/0	1/0	0/0	0/0
AD5	Familiar Mediterranean Fever	3/0	2/0	0/0	0/0	0/-1	1/0
AD6	TRAPS/CAPS	2/0	3/0	0/0	1/0	0/0	1/0
AD7	TRAPS	3/0	3/0	1/-1	1/0	0/-1	0/-1
AD8	FIRES	1/-1	0/0	0/-2	0/-2	0/0	1/0
AD9	Blau syndrome	2/-1	0/0	0/0	1/0	0/0	1/0
AD10	Sarcoidosis	2/0	2/0	0/0	1/0	0/0	2/0
AD11	SLE/connective tissue disease	3/0	3/0	0/0	0/0	1/0	1/0
AD12	Polyarthritis	1/-2	1/0	0/0	0/0	0/0	0/0
AD13	HLH	4/-2	6/0	0/0	1/0	0/0	0/0
AD14	Crohn’s disease	3/-1	2/0	1/0	1/0	0/0	1/0
AD15	Hepatic angiosarcoma	4/0	3/0	0/0	1/0	0/0	1/0

*CAPS*  Cryopyrin-Associated Periodic Syndrome, *CMV * Cytomegalovirus, *EBV  * Epstein–Barr Virus, *FIRES * Febrile Infection-related Epilepsy Syndrome, *HLH  * Hemophagocytic Lymphohistiocytosis, *MAS  * Macrophage Activation Syndrome, *(e)PDC * (Extended) Potential Diagnostic Clue, *sJIA/SD * Systemic Juvenile Idiopathic Arthritis/Still’s Disease, *SLE * Systemic Lupus Erythematosus, *SVT  * Sinus Venous Thrombosis, *TRAPS * TNF Receptor-Associated Periodic Syndrome

### Extended diagnostic procedures

Additional diagnostics identified 45 ePDC+, with serological testing confirming infections in ten children and S100A8/9 supporting sJIA/SD diagnosis in ten patients. Nine children had abnormal serological findings unrelated to the underlying diagnosis (Fig. 4c).

Advanced imaging techniques were used in 34 patients including 14 whole-body MRIs Five whole-body and nine organ-specific MRIs revealed ePDC + in eleven cases (15.3%), including six infections (meningitis, pyelonephritis, HSV encephalitis, pleuropneumonia, renal abscess and mastoiditis with empyema), two sJIA/SD (lymphadenopathy, joint effusion) and three OD cases (FIRES, SLE/mixed connective tissue disease, hepatic angiosarcoma) (Fig. 4c). Additionally, six whole-body MRIs and six organ-specific MRIs were performed in undiagnosed patients.

Of five computer tomography (CT) scans, three yielded ePDC+ (mastoiditis with empyema and sinus venous thrombosis, HSV encephalitis, sarcoidosis). Transesophageal echography revealed an ePDC + in one endocarditis case (Fig. 4c).

Genetic and histological analyses provided ePDC + in three patients: Familiar Mediterranean fever, TRAPS/CAPS, and Blau syndrome. Histology confirmed sarcoidosis and SLE (Fig. 4c).

Bone marrow biopsy was helpful in two patients: leishmaniasis and sJIA/SD complicated by macrophage activation syndrome.

## Discussion

In this nation-wide prospective surveillance study including 113 pediatric FUO cases, we analyzed clinical features and diagnostic findings across disease groups and evaluated the utility of diagnostic tests.

In our cohort, infections accounted for 36.1% of cases, with bacterial etiologies representing 26.9%, lower than the 59% reported in a systematic review [10], likely reflecting regional differences [11, 12]. Although Lyme borreliosis and cat scratch disease are increasingly reported in high resource countries likely due to improve and more accessible diagnostics [11, 13], neither was identified in our cohort. SJIA/SD was the leading cause of FUO, representing 43.1% of all diagnoses, consistent with other cohorts [14–16]. Emphasis on persistent fever as a defining symptom of various diseases varies across pediatric subspecialties and is predominantly reflected in the classification criteria for immune-mediated inflammatory diseases (IMID) e.g. for sJIA/Stills disease, KD and different autoinflammatory diseases. This differential weighting of the symptom fever may contribute to an overrepresentation of IMIDs often diagnosed by rheumatologists and underrepresentation of other diseases e.g. malignancies in FUO cohorts [17].

In our study 36.6% of patients remain undiagnosed, higher than in adults (29.9% [4]), and other pediatric cohorts (23.0% [10]), . The prevalence of FUO cases without an identified cause increased over recent decades reaching up to 67% [18]. Early preclinical diagnostic workup often directs suspicion to a specific diagnosis. Consequently, patients are referred to a specialist and are no longer hospitalized under the diagnosis of FUO [11, 18]. This may contribute to an apparent increase in the proportion of undiagnosed FUO cases within inpatient settings.

FUO management remains challenging due to the wide spectrum of rare, underlying conditions, which frequently present oligosymptomatic or with non-specific clinical features [19].

Nonetheless, history and physical examination can provide early diagnostical clues. In this instance, fever patterns can be informative and should be carefully evaluated [11, 14, 20, 21]. In our cohort, quotidian fever was common in sJIA/SD, however did not reliably discriminate from the OD group. Conjunctivitis was not exclusively reported in KD patients, consistent with previous findings, linking it to multiple FUO etiologies [19]. Pharyngitis was predominantly associated with infections, highlighting its diagnostical relevance in viral infections [13, 22]. In contrast, weight loss was a misleading sign in several patients, while it was an expected sign in Crohn’s disease and malignancies like angiosarcoma.

Exanthema, arthritis, arthralgias and myalgias were all significantly associated with sJIA. These patients also had the highest number of clinical symptoms overall, illustrating that the diagnosis is often supported by a composite presentation of symptoms [17, 23, 24].

Therefore, our observation confirms the high impact of thorough history taking and clinical examination in establishing first suspicion in children with FUO [14, 18, 21, 25].

Laboratory tests and imaging help to further identify abnormalities in pediatric FUO. To guide rational test selection various structured diagnostic strategies exist: In adults, a baseline protocol followed by two diagnostic phases was prospectively investigated [4], while for children a four-phase diagnostic process was described [26]. Our study assessed national recommendations for pediatric FUO [27], which provide a detailed but manageable set of baseline diagnostic procedures developed by extrapolating the findings from the adult FUO cohort [4]. In our cohort, patients underwent an average of 18.3 basic tests, compared with 65 per adult, likely reflecting the close monitoring protocol including regular personal visits to the recruiting hospitals performed in the later study [4, 5].

While our study revealed limited statistical differences, prior studies indicate elevated inflammatory markers (leukocytes, CRP, erythrocyte sedimentation rate (ESR)) are associated with inflammatory and infectious causes [22, 26]. In line with previous findings, ferritin was outstanding in sJIA/SD [16]. LDH values were higher among infections. In contrast, LDH was previously found mainly in children with neoplastic diseases, which is most likely due to the different cohort compositions [28].

A key aim of our analysis was to identify potentially positive and negative PDCs derived from basic diagnostical tests in order to better assess the diagnostic value of various tests in pediatric FUO.

Among microbiological test urine, blood and mucosal swap cultures were most informative yielding 12 PDCs + and 4 PDCs-. Abdominal ultrasound and echocardiography identified 45 PDCs + and 4 PDC- underscoring their important role in FUO assessment. Joint ultrasound was especially effective in sJIA/SD yielding 6 PDCs + but also identified relevant findings in 2 cases from the OD group. Furthermore, lumbar puncture and chest X-ray revealed 5 PDC+. Consistent with published data [4, 5] immunological tests, mainly antinuclear autoantibodies, produced 13 PDCs-, reinforcing recommendations to reserve antibody testing for cases with clinically suspected autoimmune pathology [19].

An average of 6.64 PDCs⁺ per patient and only 0.29 misleading PDCs⁻ (PDC⁺/PDC⁻ = 22.9) indicates that this approach, together with the used basic diagnostic tests battery, is highly effective in detecting abnormalities in pediatric FUO while generating few misleading clues.

Every child in our study had at least one PDC+ identified through the initial diagnostic approach, guiding potentially extended diagnostical tests.

Serological tests were the most frequently applied extended diagnostic procedures, resulting in a nearly equal number of confirming and misleading results, with PCR-based assays to detect viral infections such as CMV, EBV, and adenovirus infections being the most commonly performed.

Histology established the diagnosis in two cases and molecular genetic testing in three cases, underscoring their value in selected patients.

In children without helpful PDCs and compromised general condition organ-specific or whole-body MRI or PET/CT may be performed [8]. In our cohort, 14 MRIs were performed, with more than half providing relevant diagnostic information, aligning with the high diagnostical yield in another study [29, 30]. The absence of radiation exposure and nephrotoxic contrast agents makes MRI suitable for children, as recommended in a current S1 guideline for fever syndromes in children [31].

PET-CT was used in five children, providing additional diagnostic clues in three cases. Prior studies highlight the importance of correlating PET-CT findings with findings in basic diagnostic workup [32–34].

### Limitations

Due to the pseudonymized data collection, monitoring and follow-up clarification was only possible in a limited way. The exact order of diagnostic steps was not always reconstructable. Because participation of reporting hospitals was voluntary, complete capture of all incident cases cannot be ensured and selection bias cannot be excluded. Moreover, the survey design did not allow us to directly demonstrate whether extended diagnostics were initiated as a result of specific PDCs.

## Conclusions

Our study highlights the wide spectrum of underlying conditions responsible for pediatric FUO and illustrates the importance of a systematic and standardized diagnostic approach.

Physical examination and basic diagnostic procedures such as microbiological investigations, abdominal ultrasound and echocardiography revealed the majority of numerous PDCs+, while the overall rate of PDCs- remained low. This emphasizes the value of careful basic diagnostic work-up, which enhances diagnostic accuracy and prevents meaningless diagnostic tests. Abdominal ultrasound, echocardiography and bacterial cultures proved particularly effective to identify PDCs + by the basic diagnostical procedures. This enables earlier diagnosis and targeted treatment, significantly improving care and patient outcomes.

## Supplementary Information

Supplementary Material 1: Supplementary table 1; Description of data: Overview of basic and extended diagnostic procedures.

Supplementary Material 2: Supplementary table 2; Description of data: Detailed overview of PDC+ and PDC- in our cohort.

## Data Availability

The datasets used and/or analyzed during the current study are available from the corresponding author on reasonable request.

## Abbreviations

AUC: Area under the curve
CAPS: Cryopyrin–associated periodic syndrome
CMV: Cytomegalovirus
CRP: C–reactive protein
CT: Computer tomography
EBV: Epstein–Barr virus
ESR: Erythrocyte sedimentation rate
FUO: Fever of unknown origin
GPSU: German paediatric surveillance unit
HSV: Herpes simplex virus
IMID: Immune–mediated inflammatory diseases
KD: Kawasaki disease
KW: Kruskal–Wallis test
LDH: Lactate dehydrogenase
MRI: Magnet resonance imaging
(e)PDC+/: –(extended) Potentially diagnostic clue (positive/negative)
PET: CT–Positron emission tomography–computed tomography
ROC: Receiver operating characteristics
SD: Standard deviation
sJIA/SD: Systemic juvenile idiopathic arthritis/Still’s disease
SLE: Systemic lupus erythematosus
TRAPS: TNF receptor–associated periodic fever syndrome
