# Supplementary material for: Diagnostic value of basic and extended procedures in pediatric fever of unknown origin – results of a nation-wide surveillance study
Source: Mol Cell Pediatr. 2026 Jun 30;13:36. doi: 10.1186/s40348-026-00249-w (PMC13319292; doi:10.1186/s40348-026-00249-w)
Supplement: Supplementary file 1 — Supplementary Material 1: Supplementary table 1; Description of data: Overview of basic and extended diagnostic procedures. [file 40348_2026_249_MOESM1_ESM.docx]

**Supplementary material:**

**Supplementary Table 1**: Overview of basic and extended diagnostic procedures (according to (8))

| **Basic diagnostics** |
| --- |
| Hematology:  Repeated **differential blood count and peripheral blood smears**  Serum investigations:  **C-reactive protein (CRP), erythrocyte sedimentation rate (ESR)**, electrolytes, total protein, **immunoglobulins G**, M, A, and E, protein electrophoresis, **creatinine**, urea, uric acid, **ferritin,** iron, **lactate dehydrogenase** **(LDH)**, **GOT (AST), GPT (ALT)**, gamma-GT (GGT), **bilirubin**, alkaline phosphatase, **creatine kinase, coagulation parameters**, blood gas analysis, **procalcitonin**  Immunological diagnostics:  **Antinuclear antibodies (ANA)**  Microbiological diagnostics:  **Urine, cerebrospinal fluid, stool, and sputum cultures**; if necessary**, skin and mucosal swabs;** repeated **blood cultures**  Tuberculosis testing:  **Tuberculin skin test and interferon-γ release assay**  Urinalysis:  **Leukocytes, erythrocytes, protein, hemoglobin**  Stool testing:  **Occult blood, fecal calprotectin**  Imaging:  **Chest X-ray**  Ultrasound:  **Abdominal sonography**; if indicated, **sonography** **of** **joints**, pleura, and lymph nodes  Cardiac diagnostics:  **Echocardiography and electrocardiography** |
| **Extended diagnostics** |
| MRI  CT/PET-CT  Transesophageal echocardiography  Genetics  Histology  Serology  Bone marrow biopsy   - Oncological diagnostics: Catecholamines in urine, neuron-specific enolase, α1-fetoprotein, β-HCG, bone marrow puncture and biopsy - Rheumatological diagnostics: ENA, ANCA, S100 proteins - Immunological diagnostics: Immunoglobulin subclasses, blood group isoagglutinins, specific antibodies (measles, tetanus, pneumococci), repeated determination of granulocyte count, if necessary granulocyte function (oxygen radical production), if necessary flow cytometric analysis of lymphocyte and leukocyte surface markers, if necessary lymphocyte stimulation tests, complement. - Infectious disease diagnostics: Serology   - Viruses: CMV, EBV, hepatitis viruses, HIV, parvovirus B19;   - Bacteria: Bartonella henselae, Borrelia, Brucella, Campylobacter, Chlamydia, Coxiella, Ehrlichia, Francisella spp., Legionella, Leptospira, typical and atypical mycobacteria, rickettsiae, Salmonella, Spirillum minus, Tropheryma whipplei, Yersinia;   - Protozoa: Leishmania, Plasmodium, Toxoplasma gondii, Trypanosomes;   - Fungi: Aspergillus, Candida spp., Histoplasma, Cryptococcus. - Diagnostics of chronic inflammatory bowel disease: Fecal calprotectin (or fecal lactoferrin) in stool, esophagogastroduodenoscopy and ileocolonoscopy with biopsies, bowel sonography, MRI-Sellink (especially if Crohn’s disease is suspected) - Diagnostics of inflammatory hepatopathies: SMA, LKM1 and SLA antibodies, possibly liver biopsy - Diagnostics of vasculitides: ANCA testing, Doppler sonography of the carotids and aortic arch, MRI with contrast, PET, SPECT, ophthalmology (e.g. exclusion of uveitis), biopsy - Diagnostics of sarcoidosis: ACE, soluble IL-2 receptor, possibly lavage, biopsy - Diagnostics of thyroiditis: TSH, free T4/thyroxine, thyroid antibodies - Diagnostics of hereditary periodic fever syndromes: Immunoglobulin D, neutrophil alkaline phosphatase activity, urinary mevalonic acid, soluble TNF-α receptor 1, and, if necessary, molecular genetic studies: MEFV gene for FMF, Mevalonate kinase gene for HIDS, NLRP3 gene for CAPS, TNFRSF1A gene for TRAPS |

Investigations written in bold type were collected in the questionnaire.

ACE = angiotensin-converting enzyme; ALT = alanine aminotransferase; ANA = antinuclear antibodies; ANCA = antineutrophil cytoplasmic antibodies; AST = aspartate aminotransferase; β-HCG = beta-human chorionic gonadotropin; CAPS = cryopyrin-associated periodic syndromes; CMV = cytomegalovirus; CRP = C-reactive protein; CT = computed tomography; EBV = Epstein–Barr virus; ENA = extractable nuclear antigens; ESR = erythrocyte sedimentation rate; FMF = familial Mediterranean fever; GGT = gamma-glutamyl transferase; GOT = glutamate oxaloacetate transaminase; GPT = glutamate pyruvate transaminase; HIDS = hyper-IgD syndrome; HIV = human immunodeficiency virus; IL-2 = interleukin 2; LDH = lactate dehydrogenase; LKM1 = liver-kidney microsomal type 1 antibodies; MEFV = Mediterranean fever gene; MRI = magnetic resonance imaging; NLRP3 = NOD-like receptor family pyrin domain containing 3 gene; PET = positron emission tomography; PET-CT = positron emission tomography–computed tomography; SLA = soluble liver antigen antibodies; SMA = smooth muscle antibodies; SPECT = single-photon emission computed tomography; TNF-α = tumor necrosis factor alpha; TNFRSF1A = tumor necrosis factor receptor superfamily member 1A gene; TRAPS = tumor necrosis factor receptor-associated periodic syndrome; TSH = thyroid-stimulating hormone
