# Supplementary material for: Diagnostic value of basic and extended procedures in pediatric fever of unknown origin – results of a nation-wide surveillance study
Source: Mol Cell Pediatr. 2026 Jun 30;13:36. doi: 10.1186/s40348-026-00249-w (PMC13319292; doi:10.1186/s40348-026-00249-w)
Supplement: Supplementary file 2 — Supplementary Material 2: Supplementary table 2; Description of data: Detailed overview of PDC+ and PDC- in our cohort. [file 40348_2026_249_MOESM2_ESM.docx]

**Supplementary Table 2:** Detailed overview of PDC+ and PDC- in our cohort

| **Pat.** | **Diagnosis** | **PDC+**  **(Anamnesis and Clinical Findings)** | **(e)PDC+ (diagnostics)** | | | | (e)**PDC-** |
| --- | --- | --- | --- | --- | --- | --- | --- |
| Inf1 | Meningitis | Meningism | Lumbar puncture (LP): elevated cell count; **EEG, cranial MRI** | | | | Otorrhea, chest X-ray: atelectasis |
| Inf2 | CMV-meningo-encephalitis | Seizures, meningism, hepatosplenomegaly (HSM) | LP: elevated cell count, CMV; Abdominal ultrasound (AU): HSM; **Serology: CMV+** | | | |  |
| Inf3 | CMV infection |  | Urine culture: CMV PCR positive;  **Serology: CMV IgM/IgG +** | | | |  |
| Inf4 | Viral infection | Muscle pain, lymph node swelling (LNS), conjunctivitis, pharyngitis, splenomegaly | Lab: ↑CRP, ↑ESR; AU: HSM;  **Serology: Coxsackie, EBV, CMV, Mycoplasma +** | | | |  |
| Inf5 | CMV infection | Hepatomegaly, poor feeding | Blood count (BC): ↑leukocytes; Lab: ↑ESR; Urine culture: CMV-PCR positive; Sputum culture: CMV PCR + | | | | Heart murmur, Lab: **↑S100 protein** |
| Inf6 | Acute  pyelonephritis | Pain in genital region | Lab: ↑CRP; Urine: proteins; Urine culture: 10^5^ CFU/ml;  AU: LNS, splenomegaly, pyelonephritis;  **MRI: pyelonephritis** | | | |  |
| Inf7 | Pyelonephritis | Abdominal pain | BC: ↑leukocytes; Lab: ↑CRP;  Urine: leukocytes; Urine culture: E. Coli; AU: Nephritis | | | | Cervical LNS, pharyngitis, swab PCR: Enterovirus |
| Inf8 | Adenovirus infection | Sore throat, diarrhea, vomiting, pharyngitis | Lab: ↑CRP, ↑ESR;  Throat swab: Adenovirus PCR + | | | |  |
| Inf9 | Adenovirus infection | Sore throat, gastrointestinal symptoms, LNS, pharyngitis | BC: ↑leukocytes; Lab: ↑CRP;  Throat swab: Adenovirus PCR + | | | |  |
| Inf10 | Urinary tract infection |  | Lab: ↑CRP;  Urine culture: Escherichia coli | | | |  |
| Inf11 | Tonsillitis (EBV) | Sore throat, cervical LNS, pharyngitis | Lab: ↑CRP, ↑ESR; Throat swab: EBV, hemolytic streptococci group F | | | | Immunology: ANA 1:1280 |
| Inf12 | EBV infection | Cervical LNS, Pharyngitis | Lab: ↑CRP ↑ESR; **Serology: EBV** | | | |  |
| Inf13 | Coxsackie virus infection | Erythema multiforme exsudativum, pharyngitis | Lab: ↑CRP ↑ESR;  **Serology: Coxsackie IgM +, CMV, Parvovirus 19, EBV IgG+ IgM+** | | | Immunology: ANA 1:1280, borderline PR3 ANCA | |
| Inf14 | Coxsackie virus infection | Pharyngitis, aphthae | Lab: ↑CRP  **Serology: Coxsackie** | | | Stool culture: Norovirus +  Immunology: ANA 1:160 | |
| Inf15 | Endocarditis | Aortic valve disease, systolic murmur | Lab: ↑CRP; Blood culture: + (after 21 d) **TEE: endocarditis** | | | |  |
| Inf16 | Renal abscess | Flank und abdominal pain | BC: ↑leukocytes; Lab: ↑CRP, ↑ ESR; Urine: leucocytes + erythrocytes;  AU: renal abscess; **MRI: renal abscess** | | | | LP: glucose |
| Inf17 | Typhoid fever | Abdominal pain, diarrhea, inguinal LNS, splenomegaly | Lab: ↑CRP, ↑ESR; Blood culture: Salmonella typhii; Stool culture: Salmonella typhii; AU: splenomegaly, free fluid | | | |  |
| Inf18 | Yersiniosis |  | Lab: ↑CRP, ↑AST  **Serology: Yersinia IgM+ IgG+** | | | | Immunology: c-ANCA, U1-RNP |
| Inf19 | Influenza, candidemia | Pharyngitis, rhinitis | BC: ↑leukocytes; Lab: ↑CRP, ↑ESR;  Blood culture: Candida albicans; Throat swab: Hemophilus influenzae +, Influenza A +; **Serology: Candida IgM+ IgG+** | | | | Urine: protein, leukocytes |
| Inf20 | Pneumonia | Sore throat, chest pain, pharyngitis | BC: ↑leukocytes; Lab: ↑CRP, ↑ESR; Chest X-ray: infiltrates | | Swollen hands, heart murmur; AU: HSM, Echo: tricuspid + mitral insufficiency, | | |
| Inf21 | Mastoiditis, empyema, SVT | Ear and headache | Lab: ↑CRP; Wound swab: Streptococcus pyogenes; **further: CT, MRI, Histology** | | | |  |
| Inf22 | Respiratory viral infection | Loss of appetite, crackles, cough | BC: ↑leukocytes; Lab: ↑CRP;  **Serology: possible coronavirus, bocavirus, adenovirus, norovirus** | | | | Serology: Mycoplasma IgA |
| Inf23 | Parvovirus B19 infection | Exanthem, cervical LNS, myalgia | Lab: ↑CRP, ↑ESR;  **Serology: Parvovirus B19 IgM+** | | | | Urine: protein; AU: free fluid |
| Inf24 | Pleuro-pneumonia | Dyspnea | BC: ↑leukocytes; Lab: ↑CRP;  Chest X-ray: pleural effusion, **MRI: pleural effusion, infiltrate lower lobe** | | | | Exanthem, aphthae, new heart murmur, **Serology: EBV+** |
| Inf25 | Visceral Leishmaniosis | HSM | BC: ↓hemoglobin, ↓leukocytes, ↓platelets; Lab: ↑CRP; AU: HSM; **Bone marrow puncture: Leishmania spp. clonocani complex** | | | | **Serology: Parvovirus B19 IgM+** |
| Inf26 | HSV  encephalitis | Headache, dizziness, facial palsy | Lab: ↑CRP, ↑ESR; LP: Herpes simplex virus 1 PCR +; **Cranial MRI: subtle dural enhancement** | | | |  |
| S1 | sJIA/SD | Intermittent fever (IF), exanthem, arthralgia, myalgia | BC: ↑leukocytes; Lab: ↑CRP, ↑ESR, ↑ferritin; Echo: pericardial effusion;  **MRI: myositis** | | | |  |
| S2 | sJIA/SD | IF, arthralgia | BC: ↑leukocytes; Lab: ↑CRP, ↑ESR, ↑ferritin; AU: mild hepatomegaly | | | | Diarrhea, conjunctivitis |
| S3 | sJIA/SD | IF, maculopapular exanthem, LNS, HSM, arthralgia | Lab: ↑CRP, ↑ESR, ↑ferritin, ↑**S100 protein** | | | |  |
| S4 | sJIA/SD | IF, headache, bone pain, exanthem, arthritis, arthralgia, myalgia | Lab: ↑CRP, ↑ESR;  Joint ultrasound: effusion right knee | | | | **Serology: possible human herpesvirus 6** |
| S5 | sJIA/SD | IF, muscle and bone pain, fine-spotted exanthem, arthritis | BC: ↑leukocytes;  Lab: ↑CRP, ↑ESR | | | |  |
| S6 | sJIA/SD | IF, headache and bone pain, fine-spotted exanthem, cervical LNS, arthritis, arthralgia | BC: ↑platelets;  Lab: ↑CRP, ↑ESR;  AU: LNS;  Joint ultrasound: knee effusion | | | | Immunology: ANA 1:640 |
| S7 | sJIA/SD | IF, fine-spotted exanthem, cervical LNS, heart murmur, HSM | BC: ↑leukocytes, ↑platelets;  Lab: ↑CRP, ↑ESR, ↑AST/ALT;  AU: HSM; Echo: pericardial effusion | | | | Lab: ↑LDH |
| S8 | sJIA/SD | IF, chest pain, cervical and inguinal LNS, HSM, arthralgia, myalgia | Lab: ↑CRP, ↑ESR;  AU: HSM, LNS | | | | Vomiting, conjunctivitis |
| S9 | sJIA/SD | IF, bone pain, salmon-colored exanthem, arthritis, splenomegaly | BC: ↑leukocytes;  Lab: ↑CRP, ↑ESR, ↑ferritin;  AU: splenomegaly | | | |  |
| S10 | sJIA/SD | IF, salmon-colored exanthem, arthritis (<5 large joints), arthralgia | BC: ↑leukocytes; Lab: ↑CRP, ↑ESR  Joint ultrasound: right hip effusion | | | | Conjunctivitis |
| S11 | sJIA/SD | IF, bone pain, fine-spotted exanthem, arthritis (<5 joints), arthralgia | BC: ↑leukocytes, ↑platelets; Lab: ↑CRP, ↑ESR, ↑AST/ALT; Joint ultrasound: bilateral hip effusion | | | | Throat swab: Enterobacter cloacae |
| S12 | sJIA/SD | IF | BC: ↑leukocytes, ↑platelets; Lab: ↑CRP, ↑ESR | | | | Vomiting |
| S13 | sJIA/SD | IF, bone pain, cervical LNS, arthritis (<5 joints), arthralgia, myalgia | BC: ↑leukocytes;  Lab: ↑CRP, ↑ **S100 protein;**  Echo: pericardial effusion | Lab: ↑LDH; Mucosal swab: Strep B/Rhino/Parecho;  **Serology: CMV possible +;** Immunology: ANA 1:320 | | | |
| S14 | sJIA/SD | Episodic fever, refusal to walk, truncal exanthem, LNS | BC: ↑leukocytes;  Lab: ↑CRP, ↑ferritin, ↑**S100 protein** | | | | Diarrhea due to Clostridium, enterocolitis |
| S15 | sJIA/SD | IF, shoulder and knee pain, salmon-colored exanthem, arthritis, arthralgia | Lab: ↑CRP, ↑ferritin, ↑**S100 protein** | | | |  |
| S16 | sJIA/SD | IF, maculopapular exanthem, arthralgia, myalgia | Lab: ↑CRP, ↑ferritin, ↑AST/ALT, ↑**S100;**  AU: HSM; Echo: pericardial effusion | | | | Vomiting, Lab: ↑LDH |
| S17 | sJIA/SD | IF | BC: ↑leukocytes, ↑platelets;  Lab: ↑CRP, ↑ESR, ↑ **S100 protein** | | | |  |
| S18 | sJIA/SD | IF, sore throat, exanthem, cervical LNS, arthritis, splenomegaly, myalgia | Lab: ↑CRP, ↑ESR, ↑ferritin, **↑S100 protein**; AU: HSM | | | |  |
| S19 | sJIA/SD | IF, sore throat, headache, urticarial exanthem, arthritis, arthralgia, myalgia | BC: ↑leukocytes; Lab: ↑CRP, ↑ESR, ↑ferritin, **↑S100 protein;** AU: hepatomegaly | | | | Vomiting |
| S20 | sJIA/SD | Arthritis (>5 large joints) | BC: ↑leukocytes, ↑platelets;  Lab: ↑CRP, ↑ESR, ↑ferritin | | | | Continuous fever |
| S21 | sJIA/SD | IF, exanthem, arthritis (<5 large joints) | Lab: ↑CRP, ↑ferritin; AU: splenomegaly;  Joint ultrasound: effusion knee, right elbow and shoulder | | | | Immunology: ACPA 204 |
| S22 | sJIA/SD | IF, exanthem, arthritis (<5 small joints), splenomegaly | Lab: ↑CRP; Chest X-ray: infiltrates;  AU: LNS, splenomegaly;  **MRI: cervical + abdominal LNS, HSM** | | | | Urine culture: 10^5^ CFU/ml;  Immunology: ENA+Anti-dsDNA |
| S23 | sJIA/SD | Limb pain, macules during fever spike, arthritis (<5 small joints) | BC: ↑leukocytes; Lab: ↑CRP, ↑ESR, ↑ferritin; AU: mild splenomegaly;  Echo: Pericardial effusion; **MRI knee: effusion** | | | | Continuous fever; **Serology: possible Adenovirus** |
| S24 | sJIA/SD with MAS | IF, muscle and bone pain, exanthem, cervical LNS, arthritis, arthralgia | Lab: ↑CRP, ↑ESR, ↑ferritin;  AU: hepatomegaly; **bone marrow: macrophages** | Lab: ↑LDH; Immunology: ANA 1:160; **Serology: ASL/ASR/Coxsackie, Mycoplasma** | | | |
| S25 | sJIA/SD | IF, limb pain, multiform generalized exanthem, LNS, arthritis, splenomegaly, arthralgia | Lab: ↑CRP, ↑ESR, ↑ferritin ↑**S100 protein**;  AU: LNS, splenomegaly | Constipation, conjunctivitis, **Serology: EBV+CMV** | | | |
| S26 | sJIA/SD with MAS | Episodic fever, cervical and inguinal LNS, arthralgia | BC: ↑leukocytes; Lab: ↑CRP, ↑ESR, ↑ferritin, ↑**S100**; AU: hepatomegaly, joint ultrasound: abnormalities | | | |  |
| S27 | sJIA/SD | Generalized exanthem, cervical LNS, HSM, arthralgia, myalgia | Blood count: ↑platelets; Lab: ↑CRP;  Chest X-ray: pleural effusion; AU: HSM; Echo: pericardial effusion | | | | Continuous fever, crackles, dyspnea |
| S28 | Suspected sJIA | Episodic fever, exanthem, arthralgia | Blood count: ↑platelets;  Lab: ↑CRP | | | | Lab: ↑LDH;  Immunology: ANA 1:320 |
| S29 | Suspected sJIA | IF, maculopapular exanthem, arthralgia | BC: ↑leukocytes; Lab: ↑CRP, ↑ferritin ↑; Chest X-ray: pleural effusion | | | | Ear pain |
| S30 | Suspected sJIA | IF, joint and abdominal pain, exanthem | BC: ↑leukocytes, ↑platelets;  Lab: ↑CRP, ↑ESR, ↑ferritin | | | | Diarrhea; Immunology: ANA 1:100 |
| S31 | Suspected sJIA | IF, headache, abdominal pain, myalgia | BC: ↑platelets;  Lab: ↑CRP, ↑ESR, ↑ferritin | | | |  |
| AD1 | Kawasaki disease (KD) | Cervical LNS, arthritis, conjunctivitis | Lab: ↑CRP; AU: LNS | | | | Pharyngitis |
| AD2 | KD | Fine-spotted truncal exanthem, conjunctivitis, heart murmur, red dry lips | BC: ↑leukocytes, ↑platelets; Lab: ↑CRP, ↑ESR; Urine: Leucocytes, protein, erythrocytes; Echo: coronary aneurysm | | | |  |
| AD3 | Incomplete KD | Cervical LNS | BC: ↑leukocytes; Lab: ↑CRP, ↑ESR | | | | Pharyngitis |
| AD4 | KD | Maculopapular exanthem, conjunctivitis | BC: ↑platelets; Lab: ↑CRP;  Echo: coronary ectasia | | | |  |
| AD5 | Familial Mediterranean fever | Turkish origin, shoulder pain, abdominal pain, recurrent fine-spotted exanthem, nausea, vomiting | BC: ↑leukocytes; Lab: ↑CRP;  **Genetics: mutation in MEFV (Familiar Mediterranean fever gene)** | | | | Immunology: ANA 1:160 |
| AD6 | TRAPS/  CAPS | Arthritis (>5 large joints), arthralgia | Lab: ↑CRP, ↑ESR, ↑ferritin;  Joint ultrasound: joint effusion;  **Genetics: heterozygous TRAPS/CAPS** | | | |  |
| AD7 | TRAPS | Confluent exanthem, HSM | BC: ↑leukocytes;  Lab: ↑CRP, ↑ferritin;  Urine: proteins, erythrocytes; AU: HSM | Stool: adenovirus, Clostridium difficile; **Serology: CMV + parvovirus IgM;** Immunology: ANA 1:200 | | | |
| AD8 | Febrile Infection Related Epilepsy Syndrome (FIRES) | Encephalopathy, seizures | **Cerebral MRI: diffusion restriction in corpus callosum, frontotemporal edema** | Subileus; Urine: proteins; Sputum: H. influenzae, viridans streptococci; Chest X-ray: infiltrates, Echo: myocardial dysfunction | | | |
| AD9 | Blau syndrome | Papular exanthem, arthritis (<5 joints) | Joint ultrasound: ankle effusions;  **Genetics: heterozygous Arginine 578(CGT)>** | | | | Myalgia |
| AD10 | Sarcoidosis | Cervical LNS, splenomegaly | Lab: ↑IL-2 receptor, ↑ACE; AU: HSM, LNS; **PET-CT: LNS, interstitial lung changes; Histology: peritrabecular epithelioid cell granulomas** | | | |  |
| AD11 | SLE / mixed connective tissue disease | Arthralgia, cervical and axillary LNS, arthritis (>5 large joints) | Lab: ↑IgG, ↑FVIII, ↑vW antigen; Immunology: ANA 1:160, Anti-Ro-52Mb, Ds DNA +, ↓C3/C4; **MRI: enthesitis pelvis, hepatomegaly, LNS** | | | |  |
| AD12 | Polyarthritis | Arthritis (<5 joints) | Lab: ↑ESR | | | | Vomiting, nausea, abdominal LNS |
| AD13 | HLH | Headache, sore throat, generalized exanthem, seizures, cervical LNS | BC: ↓leucocytes; Lab: ↑ESR, ↑ferritin, ↑AST/ALT, ↑bilirubin, ↑LDH;  AU: splenomegaly | | | | Conjunctivitis, pharyngitis |
| AD14 | Crohn’s disease | Abdominal LNS, weight loss, aphthae | BC: ↑platelets; Lab: ↑CRP; Stool: calprotectin; AU: LNS, meteorism; **colonoscopy**: **abnormal** | | | | Helicobacter pylori gastritis |
| AD15 | Hepatic angiosarcoma | Abdominal and back pain, LNS at liver hilum, weight loss, night sweats | BC: anemia; Lab: ↑AST/ALT, ↑LDH;  AU: liver tumor;  **Histology: angiosarcoma** | | | |  |

Investigations and findings shown in bold type were collected using extended diagnostic procedures.

ALT = Alanine Aminotransferase, ANA = Antinuclear Antibodies, ANCA = Anti-Neutrophil Cytoplasmic Antibodies, AST = Aspartate Aminotransferase, AU = Abdominal Ultrasound, BC = Blood Count, CAPS = Cryopyrin-Associated Periodic Syndrome, CFU = Colony Forming Units, CMV = Cytomegalovirus, CRP = C-Reactive Protein, CT = Computed Tomography, dsDNA = double-stranded DNA, EBV = Epstein–Barr Virus, EEG = Electroencephalography, ENA = Extractable Nuclear Antigens, ESR = Erythrocyte Sedimentation Rate, HLH = Hemophagocytic Lymphohistiocytosis, HSM – Hepatosplenomegaly, IF = Intermittent Fever, LDH = Lactate Dehydrogenase, LNS = Lymph Node Swelling, LP = Lumbar Puncture, MAS = Macrophage Activation Syndrome, MRI = Magnetic Resonance Imaging, PCR = Polymerase Chain Reaction, PDC = Potential Diagnostic Clue, PET-CT = Positron Emission Tomography–Computed Tomography, sJIA/SD – Systemic Juvenile Idiopathic Arthritis/Still’s Disease, SLE = Systemic Lupus Erythematosus, SVT = Sinus Venous Thrombosis, TEE = Transesophageal Echocardiography, TRAPS = TNF Receptor-Associated Periodic Syndrome, U1-RNP = U1 ribonucleoprotein
